# Reduced Respiratory Sinus Arrhythmia in Infants with the *FMR1* Premutation

**DOI:** 10.3390/ijms26052186

**Published:** 2025-02-28

**Authors:** Abigail Chase, Lisa Hamrick, Holley Arnold, Jenna Smith, Rachel Hantman, Kaitlyn Cortez, Tatyana Adayev, Nicole D. Tortora, Alison Dahlman, Jane Roberts

**Affiliations:** 1 School of Medicine, University of South Carolina, Columbia, SC 29209, USA; abigail.chase@uscmed.sc.edu; 2 Department of Psychology, University of South Carolina, Columbia, SC 29208, USA; rague@mailbox.sc.edu (L.H.); holley.pitts.arnold@sc.edu (H.A.); jennags@email.sc.edu (J.S.); rhantman@email.sc.edu (R.H.); kbcortez@email.sc.edu (K.C.); adahlman@mailbox.sc.edu (A.D.); 3 Carolina Autism and Neurodevelopment Research Center, University of South Carolina, Columbia, SC 29208, USA; 4 Department of Human Genetics, New York State Institute for Basic Research in Developmental Disabilities, New York, NY 10314, USA; tatyana.adayev@opwdd.ny.gov (T.A.); nicole.d.tortora@opwdd.ny.gov (N.D.T.)

**Keywords:** *FMR1* premutation, CGG repeat length, autonomic nervous system, respiratory sinus arrhythmia, interbeat interval

## Abstract

The fragile X premutation (FXpm) is caused by a CGG repeat expansion on the *FMR1* gene. In adults, FXpm is linked with autonomic nervous system (ANS) dysfunction and impairment is associated with CGG repeat length. Given scant infancy research, we examined ANS functioning, via respiratory sinus arrhythmia (RSA) and interbeat interval (IBI), in 82 FXpm and neurotypical infants and their associations with CGG repeats. FXpm infants exhibited lower RSA but no IBI differences. There were no associations between ANS functioning and CGG repeat length. These findings identify an ANS biomarker consistent with the emerging pediatric phenotype in FXpm.

## 1. Introduction

Fragile X messenger ribonucleoprotein 1 (*FMR1*)-associated disorders include multiple conditions that arise from cytosine–guanine–guanine (CGG) trinucleotide repeat expansions in the 5′UTR of the *FMR1* gene located on the X chromosome, which encodes for the *FMR1* protein (FMRP). FMRP plays a role in neuronal expansion, plasticity, and translation regulation. It is widely expressed, including in the brain and in the reproductive system where it contributes to fertility regulation. *FMR1* mutations are categorized by the number of CGG repeats. Fragile X syndrome (FXS) occurs when there are more than 200 CGG repeats, leading to the silencing of the *FMR1* gene, with deficits in the FMRP. FXS is the most common heritable form of intellectual disability [1,2] and has been the focus of extensive research for nearly 50 years. Individuals with 55 to 200 CGG repeats have the *FMR1* premutation (FXpm), which is associated with subtle, but measurable, impacts on health and psychological functioning [3,4,5,6,7,8]. In the United States, while FXS is rare, affecting 1:8000 females and 1:5000 males, the FXpm is common, affecting 1:151–291 females and 1:468–845 males [9,10,11].

Research on the FXpm has accelerated over the past 20 years given increasing evidence that a subgroup of individuals with the FXpm are meaningfully affected. However, most of this work has focused on adults; research on the pediatric phenotype of FXpm has only recently started to emerge and indicates developmental delays or impairments that are detectable within the first years of life [12,13,14]. Early identification and treatment aimed at reducing severity or preventing impairment is critical to these children. While many individuals with the FXpm may be unaware of their genetic status due to lack of population screening, increased prenatal and newborn genetic screening [15] has resulted in earlier and more widespread identification of the FXpm, which escalates the need for research to identify the underlying mechanisms associated with impairment to direct treatment. This is particularly important during the infant and preschool years when intervention is known to be most effective [16,17,18,19,20].

Despite the importance of understanding FXpm in infancy, research is scant. The FXpm phenotype has been primarily drawn from research with adults, showing significant differences between individuals with the FXpm and those who are not carriers [21]. While the phenotypic presentation of FXpm is generally less severe than that of FXS, those with the FXpm are clearly at an increased risk for multiple conditions. These are collectively referred to as fragile X premutation-associated conditions (FXPAC) [22]. FXPAC includes fragile X-associated tremor/ataxia syndrome (FXTAS), a late-onset neurodegenerative disorder that affects approximately 40% of male carriers and 15% of female carriers [23,24], and fragile X primary ovarian insufficiency (FXPOI), which leads to early menopause affecting 20–30% of female carriers [25]. A group of neuropsychiatric symptoms and disorders, collectively referred to as fragile X-associated neuropsychiatric disorders (FXAND), includes anxiety, mood disorders, attentional difficulties, executive function deficits, and pragmatic social language challenges [26,27].

The mechanistic underpinnings of the FXpm phenotype in adults are largely not well-characterized; however, evidence suggests multiple interacting variables, including environmental, psychological, molecular and genetic factors. Environmental stress (e.g., parenting status, life events) has been linked to difficulties with executive function and memory in mothers with the FXpm [28], while higher education confers neuroprotection for these women [29,30]. Genetic factors that affect the somatic instability of the CGG repeat number may contribute to the variable penetrance for FXPAC and disease pathophysiology. Moreover, the pathophysiology in FXPAC includes dysregulation in several molecular mechanisms, such as elevated mRNA levels from corresponding elevation in transcription, repeat association non-AUG translation resulting in aggregation of polyglycine-containing protein, glial dysregulation, co-transcriptional R loop formation, altered expression of long noncoding RNAs, and neurotoxic levels of *FMR1* mRNA [31,32,33,34,35,36,37,38].

CGG length has also been clearly implicated as a mechanistic variable contributing to the FXpm phenotype in adults. While longer CGG repeat length has been implicated with more severe impairment in the FXpm [14,39], multiple studies in adult women have identified a curvilinear pattern with increased impairment associated with mid-range (~80–120) CGG length [40,41,42]. Women with CGG repeats in this mid-range face the highest risk for earliest onset of FXPOI [43] and the highest risk of developing FXTAS [44]. The elevated risk for individuals with mid-range CGG length alleles is complex; however, research in adults indicates that variation in CGG length interacts with environmental stress exposure, education, sex, and age [28,30,45].

To advance the identification of factors that confer elevated risk for impairment in the FXpm, researchers have investigated the role of the autonomic nervous system (ANS), which governs responses to challenges in the environment [46] and comprises the sympathetic nervous system (SNS; “fight-or-flight”) and the parasympathetic nervous system (PNS; “rest-and-digest”). ANS function can be measured non-invasively to derive two important elements: interbeat interval (IBI), the time between successive heartbeats that is influenced by both PNS and SNS, and respiratory sinus arrhythmia (RSA), the cardiac indicator that isolates the PNS and the vagal impact on cardiac activity [47,48]. Importantly, RSA has been conceptualized as a peripheral marker of emotion regulation capacity, which makes its study in clinical populations at risk for autism or affective disorders highly relevant [49].

ANS function in *FMR1* conditions has primarily been studied in individuals with FXS and adult women with the FXpm. In FXS, ANS dysfunction has long been proposed as a key mechanism contributing to the FXS phenotype [50,51]. Research consistently indicates that, relative to neurotypical (NT) controls, children and young adults with FXS exhibit reduced RSA (i.e., decreased PNS activity and increased SNS activity) and shorter IBI at baseline [52,53,54,55,56,57,58]. This pattern of diminished parasympathetic activity and heightened sympathetic activity emerges within the first years of life and is predictive of autism spectrum disorder (ASD) and anxiety in young children with FXS [52,53,54]. Likewise, research with women with the FXpm has shown reduced baseline RSA that mediates pragmatic language deficits [27]. However, RSA was not associated with anxiety and depression in women with the FXpm, implying that only a subset of phenotypic FXpm features aligning with ASD and ASD-related conditions may be affected by RSA [27]. No studies have examined ANS function in infants or young children with the FXpm. Given evidence of low RSA predicting ASD in infants and young children without *FMR1* conditions [59,60,61] and the emerging pediatric phenotype that includes sensory processing disruption, poor eye contact, social inhibition, and social communication differences [13,14,62], all of which are associated with ASD, investigation advancing ANS function in infants with the FXpm is critical.

Despite research demonstrating CGG repeat length within the FXpm range is linked to disruptions in regulatory pathways potentially influencing ANS function [62], the molecular genetic underpinnings of ANS function in the FXpm have not been well-studied. Evidence with adult women with the FXpm suggests that longer CGG repeat length and elevated mRNA are correlated with higher baseline RSA [5]. However, no studies have examined the relationship of ANS function to CGG repeat length or other molecular or genetic variables in children with FXpm. Thus, we know very little about the relationship of ANS dysfunction to molecular and genetic variables, especially among the pediatric population. Investigating whether and how molecular or genetic variation in the FXpm are associated with ANS function will advance understanding of biological mechanisms that influence the FXpm human phenotype to develop interventions.

The focus of this study is to advance understanding of ANS function with a non-biased sample (e.g., not identified due to clinical referral) of FXpm infants. Drawing from research in FXS and adults with the FXpm, we hypothesized that infants with the FXpm will exhibit ANS dysfunction (i.e., reduced RSA and shorter IBI) that will implicate concurrent or future impairment. We also hypothesized that CGG repeat length will be associated with ANS function. This study will expand the extant literature by linking ANS function with molecular or genetic markers in FXpm infants, thus further elucidating the FXpm pediatric phenotype.

We addressed two primary research questions:

1. Do baseline RSA and IBI differ in 12-month-old infants with the FXpm compared to NT controls?
2. Is there a relationship between CGG repeat length and baseline RSA or IBI in 12-month-old infants with the FXpm?

## 2. Results

**Figure 1:**
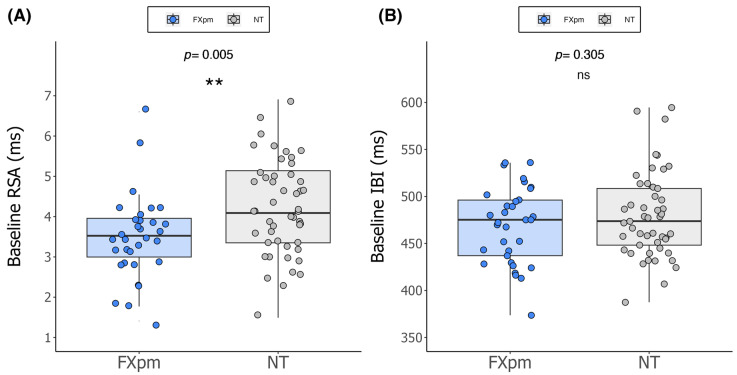
Baseline cardiac activity as a function of group. Note. Distribution of (**A**) respiratory sinus arrhythmia (RSA) and (**B**) interbeat interval (IBI) in fragile X premutation (FXpm) and neurotypical (NT) groups. ** *p* ≤ 0.01, ns = non-significant (*p* > 0.05).

Analyses indicated that the FXpm and NT groups did not significantly differ on chronological age (*t*(80) = −1.03, *p* = 0.306) or sex distribution (χ^2^(1) = 0.812, *p* = 0.368). Figure 1 indicates the distribution of cardiac values for FXpm and NT groups. Mean baseline RSA was significantly lower in FXpm infants compared to NT infants (*t*(79) = −2.922, *p* = 0.005, *d* = −0.66), with a medium to large effect size [63]. Mean baseline IBI did not significantly differ (*t*(80) = −1.302, *p* = 0.305, *d* = −0.232), with a small effect.

**Figure 2:**
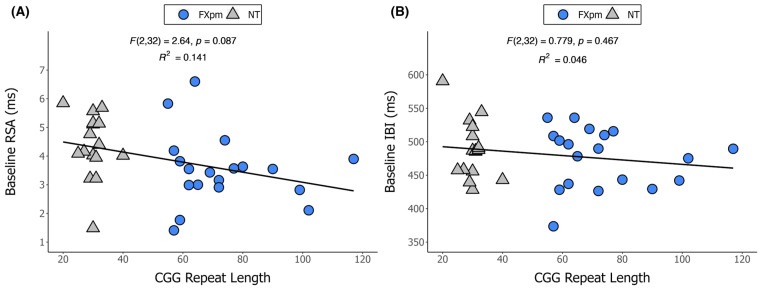
CGG repeat length as a function of baseline cardiac activity. Note. CGG repeat length as a function of (**A**) respiratory sinus arrhythmia (RSA) or (**B**) interbeat interval (IBI) in fragile X premutation (FXpm) and neurotypical (NT) groups.

Figure 2 depicts the relationship between cardiac values and CGG repeat length. The overall linear regression models using CGG repeat and CGG repeat^2^ predicting RSA (*F*(2,32) = 2.64, *p* = 0.087) and IBI (*F*(2,32) = 0.779, *p* = 0.467) were not significant, with little to no variance in the ANS outcomes explained (*R*^2^*_RSA_* = 0.141; *R*^2^*_IBI_* = 0.046). For RSA and IBI, neither CGG repeat (RSA: *B* = −0.04, *p* = 0.262; IBI: *B* = −0.882, *p* = 0.502) nor CGG repeat^2^ (RSA: *B* = 0.000, *p* = 0.525; IBI: *B* = 0.005, *p* = 0.661) were significant predictors.

Follow-up regression models with only the FXpm group were conducted. Results were similar. For RSA and IBI, neither CGG repeat (RSA: *B* = −0.055, *p* = 0.747; IBI: *B* = −1.861, *p* = 0.762) nor CGG repeat^2^ (RSA: *B* = 0.000, *p* = 0.791; IBI: *B* = 0.010, *p* = 0.790) were significant predictors. To confirm the lack of significance was not due to insufficient power within the FXpm sample, Pearson correlations were evaluated. The analysis revealed a non-significant relationship between RSA and CGG repeat (*r*(19) = −0.141, *p* = 0.564) and between RSA and CGG repeat^2^ (*r*(19) = −0.134, *p* = 0.586). The analysis also confirmed a non-significant relationship between IBI and CGG repeat (*r*(19) = −0.092, *p* = 0.707) and between IBI and CGG repeat^2^ (*r*(19) = −0.085, *p* = 0.730).

## 3. Discussion

The FXpm is a prevalent genetic variant associated with elevated likelihood of cognitive, psychological, and health problems in adulthood [4,6,7,64,65,66,67]. Research on the pediatric phenotype of the FXpm is increasing given evidence of developmental delays and clinical difficulties that emerge early in development [13,14,62,68]. The mechanistic underpinnings of these early emerging pediatric FXpm phenotypic features are largely unknown, though evidence from research focused on FXS or adults with the FXpm suggests environmental and molecular genetic factors [14,30,52,53]. The present study contributes to the field by identifying an ANS biomarker, RSA, which likely influences concurrent or future impairments in the FXpm pediatric phenotype.

We report reduced RSA in 12-month-old infants with the FXpm contrasted against age- and sex-matched NT controls. This finding is unprecedented, as this is the first study to identify biomarkers as a putative mechanism associated with the FXpm pediatric phenotype in infants this young, as studies to date have focused on clinical symptoms or reported biomarkers at a later age [14]. Previous studies have linked lower RSA/ANS dysfunction to behavioral phenotypes and disorders associated with adult women with the FXpm and/or have identified common behavioral phenotypes in FXpm, but no research has examined RSA/ANS dysfunction with FXpm infants. By identifying lower RSA in FXpm infants, our work has extended the literature to document a potential biological mechanism that connects ANS (dys)function to behavior in the FXpm pediatric phenotype. Our finding of lower RSA aligns with research in related clinical groups that link reduced RSA to difficulties in social and emotion regulation [61,69]. It also supports emerging evidence of social and sensory processing difficulties in 12-month-old infants with the FXpm [14] and lower social communication competence in preschoolers with the FXpm [13], which are behaviors that have been linked to lower RSA/ANS dysfunction in other groups [59,60,61,70], thus connecting these behavioral phenotypes with lower RSA in FXpm infants. Our finding is also consistent with the high rates of FXAND diagnoses in children with the FXpm, including anxiety (82.0%), ADHD (66.5%), and ASD (32.8%) [68], as these disorders are also associated with lower RSA/ANS dysfunction [52,59,60,61,70]. These current results also complement our previous work showing reduced RSA at 24 months predicts increased ASD symptoms at 36 months for both NT controls and children with FXS [52]. Likewise, lower RSA is observed in adult females with the FXpm and is linked to discrete elements of the phenotype, including pragmatic language [5,27].

The identification of lower RSA in the FXpm at 12 months is remarkable, as most research has reported RSA differences at older ages even in clinical groups with a more severe clinical profile. For instance, in ASD samples, no studies have reported lower RSA during infancy; 18 months of age has been the earliest age to date reported with reduced RSA [71]. In a sample of infants with FXS, our team reported reduced RSA that emerged by 12 months and was associated with increased autism symptom severity [54]. In FXS males, we also identified a trajectory of RSA that became increasingly lower than NT controls from 3 to 83 months of age which predicted later autism symptoms [52]. Given the milder phenotype of the FXpm, we anticipated that lower RSA in this small sample of infants might not be detectible at this early age. The fact that RSA is, in fact, lower than the NT controls by 12 months of age with a medium to large effect size in this relatively small sample suggests that the *FMR1* gene has a strong influence on RSA in the FXpm and could account for at least a subset of the phenotypic features.

In contrast to reduced RSA in the infant FXpm group, IBI did not differ across the groups. This was not entirely unexpected given the unique relationship that RSA has to social communicative and affective aspects of behavior, which is consistent with the emerging FXpm pediatric phenotype. Given the link of RSA to these facets of behavior that also align with the ASD phenotype, most research on ANS function in ASD has focused on RSA, with no studies reporting IBI during infancy. However, IBI has been examined in infants with FXS, and evidence from our team previously identified shorter IBI (e.g., faster heart rate) across infancy that was associated with elevated likelihood of ASD in FXS when adopting both cross-sectional [54] and longitudinal approaches [53]. Thus, we hypothesized that, despite their early age and mild phenotype, IBI might be shorter in the FXpm infants. The lack of group differences could be attributed to developmental effects, as evidence suggests that lower RSA emerged earlier than shorter IBI at 24 and 29 months of age, respectively, in FXS [52]. In support of this hypothesis, we report a small effect size estimate in the current analyses which, despite non-significant results, supports continued examination of IBI in infants with the FXpm, as differences could emerge later in development. The lack of group differences in IBI could also be attributed to the discrete functions of IBI vs. RSA (e.g., anxiety versus ASD) that we and others have reported calling for continued surveillance with larger samples.

Despite both RSA and IBI representing ANS function, consistent evidence from our group and others has documented unique contributions across these two indices that may play an important role in specific physiological functions, individual differences, and methodological factors. Other neurodevelopmental disorders such as ASD can help contextualize the autonomic function and developmental outcomes in *FMR1* conditions. For example, we found that lower RSA across 3 to 83 months of age emerged at 24 months of age and predicted elevated ASD symptoms, while shorter IBI emerged at 29 months of age and predicted increased symptom severity of anxiety in males with FXS [52], suggesting different roles for RSA versus IBI in relation to varying outcomes. In addition, a different study found that shorter IBI across infancy predicted ASD diagnoses in males and females with FXS, suggesting potential sex effects [53]. There are no studies using IBI in the FXpm pediatric literature. Evidence suggests nuanced relationships between RSA and the clinical phenotype in adult women with the FXpm. Klusek and colleagues reported that lower RSA is associated with only a subset of phenotypic features (e.g., pragmatic language) but not across all features (e.g., anxiety, depression) in adult females with the FXpm, despite these relationships existing for neurotypical controls [5,27]. We found significant differences in RSA within this FXpm sample; however, the FXpm population is inherently heterogeneous, with variability driven by numerous factors, including age, sex, CGG repeat length, and environmental factors. These sources of individual differences may contribute to variations in autonomic function, highlighting the need for further research to disentangle the specific mechanisms driving RSA differences within this population using larger samples to investigate the impact of these variables on ANS function in the pediatric FXpm phenotype. Findings from this study and others clearly establish that the ANS is a complex neurobiological system driven by molecular and genetic factors, as well as individual differences and environmental variables that, collectively, influence multiple aspects of human functioning.

Our finding that CGG repeat length was not associated with RSA or IBI suggests that other molecular or genetic variables are involved during this early developmental period (e.g., mRNA, FMRP, activation ratio in females). The relationship of CGG repeat length to the pediatric phenotype has been reported in only one study, with longer CGG repeat length associated with higher overall developmental scores despite the FXpm group having similar developmental scores to the NT group [14]. In contrast, CGG repeat length was not associated with language, behavior, or autism features in that study, suggesting that the relationship of genetic or molecular variables to the pediatric phenotype of FXpm is complex, as has been established in studies of adults with the FXpm [5,27]. Due to the varying CGG repeat length within the premutation range, it is possible that autonomic function can differ within subgroups of lower-range, mid-range, or high-range repeats. As noted earlier, evidence suggests that mid-range CGG repeats may represent elevated risk in adults [40,41,72]; yet, our results do not support a relationship between linear or curvilinear CGG repeat length in infancy. There are several potential explanations for this finding. First, the sample may be too small to detect these relationships. Second, the CGG repeat length of those with the FXpm is not evenly distributed across the full range with a mean of 73.3, range between 55 and 117, and only 26% (5 out of 19) falling in the mid-range of 80–120. Thus, the distribution of CGG repeat length in our sample might have influenced the findings. Third, developmental factors might have affected our findings, as the relationship between CGG repeat length and developmental scores in the Wheeler (2016) study occurred at the latest age point, which was 35 months or older for most children, while most of our sample is 12 months old [14].

While this study includes the largest non-clinically referred sample of 12-month-old infants with the FXpm published to date, the sample size is limited with only 33 infants with the FXpm to examine ANS function and only 19 with CGG repeat data to determine the relationship of ANS function to CGG repeat length. This small sample precludes confidence in our power to detect group differences in IBI and to examine individual differences that could influence ANS function (e.g., age, sex, developmental level, intervention, parental stress). It is imperative to replicate this study with a larger sample size to identify trends in ANS (dys)function, accounting for potential linear or non-linear effects of CGG repeats on RSA and IBI that influence the phenotype. Also, our sample is not longitudinal, which is critical for identifying important developmental trajectories to provide comprehensive understanding of physiological indices throughout childhood. We were also limited in the molecular and genetic variables available to analyze, which restricted our analyses to CGG repeat length in a subgroup. Future research should include larger samples that are tracked longitudinally, as well as include multiple biomarkers and a comprehensive clinical battery to systematically test hypotheses regarding putative mechanisms associated with the FXpm pediatric phenotype. Additionally, an important direction for future research involves exploring the clinical implications of these findings, particularly in relation to early identification and intervention for infants with the *FMR1* premutation. Given evidence of early ANS differences in this population, investigating how this biomarker can inform screening protocols and tailored early intervention strategies could significantly enhance developmental outcomes (e.g., treatment to elevate RSA). Understanding the potential for early detection may also help allow for more precise monitoring and support for infants at risk for neurodevelopmental challenges associated with the FXpm.

Despite these limitations, this study contributes critical evidence to the field given that the FXpm is a common genetic variant that is increasingly being diagnosed in infants through prenatal and newborn screening [15]. Once thought to be a condition primarily associated with elevated risk of transmission and expansion to FXS in offspring, research has now clearly established that a subgroup of individuals with the FXpm are affected by elevated social, cognitive, and affective conditions including anxiety, stress, ADHD, and ASD [26,68]. There is a tremendous gap in the field regarding the pediatric phenotype of the FXpm, particularly during the infant developmental period, and in the investigation of mechanisms that influence function. We addressed these gaps by identifying reduced RSA at 12 months of age as a biomarker associated with the pediatric clinical profile of the FXpm.

## 4. Materials and Methods

### 4.1. Participants

This cross-sectional study included 82 twelve-month-old infants (FXpm: *n* = 33; NT: *n* = 49) drawn from two longitudinal studies of early development in *FMR1* conditions (R01MH090194; R01HD106652). Inclusion/exclusion criteria were consistent for both longitudinal studies including full-term birth and hearing and vision within normal limits. Recruitment for the FXpm sample varied across studies, with one study (R01MH090194) utilizing social media and collaboration amongst FX researchers as the primary method with parents confirming FXpm status via medical or genetic records that typically, but not universally, included CGG repeat length in the record. In contrast, the second study (R01HD106652) primarily recruited the FXpm through a collaboration with the Institute for Basic Research Specialty Clinical Laboratories (IBR SCL) that processed prenatal samples and collaborated with genetic counselors who informed eligible families of our study. None of the participants in this study were identified through clinical referral, so this represented a sample that is not biased towards more severe clinical symptoms as has been reported in the literature [68].

All FXpm infants with baseline heart activity were included in analyses for the first research question, and FXpm infants with genetic records that included CGG repeat number were included in analyses for the second research question (*n* = 19; 11 m, 8f). For participants with multiple assessments, the time point in closest proximity to the 12-month assessment was prioritized, as that age maximized the amount of data available while still retaining a focus on the early infant developmental period when phenotypic differences in the FXpm have emerged [14]. The NT infants were matched on age and sex to the FXpm group to reduce potential confounding factors. NT children were recruited from the community and were excluded if there was a family history of fragile X, autism, or a related diagnosis. NT infants were also excluded if they demonstrated developmental delays on either the Bayley Scales of Infant and Toddler Development (BSID), 4th Edition [73], or Mullen Scales of Early Learning (MSEL) [74] developmental assessments (standard scores ≤ 70). Genetic testing of NT children was only completed as a preliminary biological marker for a subset of children in one study (R01MH090194) and was completed for all NT participants in the second study (R01HD106652), so NT infants with CGG repeat length data were included in the second research question (*n* = 16; 9 m, 7f). Demographic characteristics are included in Table 1.

### 4.2. ANS Function

Baseline ANS function was derived based on heart activity measured using a telemetry-based monitor that sampled the ECG signal at 1024 Hz via two electrodes placed on the infant’s chest [75,76] while infants watched a 3 min Baby Einstein video containing sound effects and music but no spoken language. From extracted data, the IBI was inspected to edit arrhythmias and artifacts using CardioEdit V7 software and excluded if >10% of data had to be edited [77]. In this sample, the average of edited IBI data using CardioEdit software was 0.63% of IBI values per file, reflecting highly accurate signal detection and a low proportion of files that were edited. RSA was extracted via the transformed variance into its natural logarithm from the edited IBI data using CardioBatchPlus V7 software [78]. In the software algorithm, the associated filter, 0.24–1.04 Hz was applied; mean IBI and mean RSA values were derived and included as dependent variables. Descriptives are listed in Table 2.

### 4.3. CGG Repeat Length

Diagnosis of FXpm and determination of CGG repeat length were confirmed through genetic reports of *FMR1* analyses either through parental sharing of medical records or through participation in the IBR SCL. For participants who underwent prenatal screening at the IBR SCL, the prenatal clinical report that confirmed FXpm diagnostic status was shared following parental authorization. To quantify CGG repeat length, *FMR1 CGG* repeat-primed PCR was performed on genomic DNA using AmplideX^®^ PCR/CE *FMR1* Kit [79] as previously described [80,81,82]. Descriptives are listed in Table 2.

### 4.4. Procedure

Assessments occurred at participants’ homes or in a family-friendly research laboratory. Baseline cardiac activity was the initial assessment activity typically completed after parental consent with developmental assessments occurring after the baseline cardiac data were collected. Assessments were prioritized for mid-morning to maximize infant attention and to control for the potential influences of circadian rhythm.

### 4.5. Statistical Plan

Statistical analyses were completed using SPSS 27 [83]. Preliminary analyses were run, including descriptive analyses for all variables of interest. Group differences in chronological and biological sex were evaluated, and correlations between all study variables were conducted. These analyses were critical to confirm group differences that could contribute to our primary research question analyses and interpretations. To address our first research question regarding whether group differences in baseline cardiac values were evident, independent *t*-tests were conducted. To address our second research question, we ran multiple regression models to evaluate the relationship between cardiac values and CGG repeats. Either RSA or IBI were included as the dependent variable. The independent variables were CGG repeat and CGG repeat^2^. CGG repeat^2^ was included to probe the curvilinear (quadratic and non-linear) patterns seen in the extant adult literature. Analyses were run within the full sample and then with the FXpm sample only. Assumptions of multiple linear regression analyses were met. Detected outliers were evaluated using Cook’s distance [84]. Cook’s distance greater than 4/n were calculated to assess influential outliers. Cases with a distance above the threshold were removed for each analysis; however, statistical significance remained consistent, thus reported results are for the full sample size. Due to a relatively small sample of FXpm infants, we completed Pearson bivariate correlations to confirm the regression results were not limited due to a lack of power.

## Figures and Tables

**Table 1:** Sample demographic characteristics.

	FXpm (*n* = 33)	NT (*n* = 49)	Total (*n* = 82)
Age (in months)			
Mean (SD)	12.1 (1.5)	11.8 (1.4)	11.9 (1.4)
Range	8.7–14.5	8.8–13.9	8.7–14.5
Sex			
Male	19 (57.6%)	33 (67.4%)	52 (63.4%)
Female	14 (42.4%)	16 (32.7%)	30 (36.6%)
Race			
White	28 (84.8%)	38 (77.6%)	66 (80.5%)
Black/African Am.	0 (0%)	6 (12.2%)	6 (7.3%)
Asian	1 (3.0%)	0 (0%)	1 (1.2%)
More than one race	4 (12.2%)	5 (10.2%)	9 (11.0%)
Ethnicity			
Hispanic	2 (6.1%)	5 (10.2%)	7 (8.5%)
Non-Hispanic	31 (93.9%)	44 (89.8%)	75 (91.5%)
MSEL ELC ^a^			
N	9	28	37
Mean (SD)	93.9 (9.5)	98.7 (14.8)	97.5 (13.7)
Range	82–111	71–134	71–134
BSID Cognitive SS ^b^			
N	24	21	45
Mean (SD)	93.1 (13.6)	97.6 (8.5)	95.2 (11.6)
Range	70–115	80–110	70–115

^a^ MSEL ELC = Mullen Scales of Early Learning Composite Standard Score. ^b^ BSID Cognitive SS = Bayley Scales of Infant Development, 4th Edition, Cognitive Scale Standard Score.

**Table 2:** Sample descriptives.

	FXpm (*n* = 33)		NT (*n* = 49)	
	M (SD)	Range	M (SD)	Range
CGG Repeat Length ^a^	73.3 (17.4)	55–117	29.9 (4.1)	20–40
RSA	3.5 (1.0)	1.4–6.6	4.2 (1.2)	1.5–6.9
IBI	469.3 (40.5)	373.7–535.9	479.4 (45.3)	387.5–594.7

^a^ Available for 19 participants with FXpm and 16 NT controls. For those with differing CGG repeats, the highest number of repeats was used for analysis.

## Data Availability

The data presented in this study are available on request from the corresponding author due to privacy reasons given the sensitive nature of the study.

## Abbreviations

The following abbreviations are used in this manuscript:

ANS	Autonomic nervous system
ASD	Autism spectrum disorder
BSID	Bayley Scales of Infant and Toddler Development
CGG	Cytosine–guanine–guanine
FMR1	Fragile X messenger ribonucleoprotein 1
FMRP	FMR1 protein
FXAND	Fragile X-associated neuropsychiatric disorders
FXPAC	Fragile X premutation associated conditions
FXpm	Fragile X premutation
FXS	Fragile X syndrome
FXTAS	Fragile X-associated tremor/ataxia syndrome
IBI	Interbeat interval
IBR SCL	Institute for Basic Research Specialty Clinical Laboratories
MSEL	Mullen Scales of Early Learning
NT	Neurotypical
PNS	Parasympathetic nervous system
RSA	Respiratory sinus arrhythmia
